# Low-Cost Consumer-Based Trackers to Measure Physical Activity and Sleep Duration Among Adults in Free-Living Conditions: Validation Study

**DOI:** 10.2196/16674

**Published:** 2020-05-19

**Authors:** Laurent Degroote, Gilles Hamerlinck, Karolien Poels, Carol Maher, Geert Crombez, Ilse De Bourdeaudhuij, Ann Vandendriessche, Rachel G Curtis, Ann DeSmet

**Affiliations:** 1 Department of Movement and Sports Sciences Ghent University Ghent Belgium; 2 Department of Experimental Clinical and Health Psychology Ghent University Ghent Belgium; 3 Research Foundation Flanders Brussels Belgium; 4 Department of Communication Studies University of Antwerp Antwerp Belgium; 5 School of Health Sciences University of South Australia Adelaide Australia; 6 Department of Public Health and Primary Care Ghent University Ghent Belgium; 7 Department of Clinical and Health Psychology Université Libre de Bruxelles Brussels Belgium

**Keywords:** fitness trackers, mobile phone, accelerometry, physical activity, sleep

## Abstract

**Background:**

Wearable trackers for monitoring physical activity (PA) and total sleep time (TST) are increasingly popular. These devices are used not only by consumers to monitor their behavior but also by researchers to track the behavior of large samples and by health professionals to implement interventions aimed at health promotion and to remotely monitor patients. However, high costs and accuracy concerns may be barriers to widespread adoption.

**Objective:**

This study aimed to investigate the concurrent validity of 6 low-cost activity trackers for measuring steps, moderate-to-vigorous physical activity (MVPA), and TST: Geonaut On Coach, iWown i5 Plus, MyKronoz ZeFit4, Nokia GO, VeryFit 2.0, and Xiaomi MiBand 2.

**Methods:**

A free-living protocol was used in which 20 adults engaged in their usual daily activities and sleep. For 3 days and 3 nights, they simultaneously wore a low-cost tracker and a high-cost tracker (Fitbit Charge HR) on the nondominant wrist. Participants wore an ActiGraph GT3X+ accelerometer on the hip at daytime and a BodyMedia SenseWear device on the nondominant upper arm at nighttime. Validity was assessed by comparing each tracker with the ActiGraph GT3X+ and BodyMedia SenseWear using mean absolute percentage error scores, correlations, and Bland-Altman plots in IBM SPSS 24.0.

**Results:**

Large variations were shown between trackers. Low-cost trackers showed moderate-to-strong correlations (Spearman *r*=0.53-0.91) and low-to-good agreement (intraclass correlation coefficient [ICC]=0.51-0.90) for measuring steps. Weak-to-moderate correlations (Spearman *r*=0.24-0.56) and low agreement (ICC=0.18-0.56) were shown for measuring MVPA. For measuring TST, the low-cost trackers showed weak-to-strong correlations (Spearman *r*=0.04-0.73) and low agreement (ICC=0.05-0.52). The Bland-Altman plot revealed a variation between overcounting and undercounting for measuring steps, MVPA, and TST, depending on the used low-cost tracker. None of the trackers, including Fitbit (a high-cost tracker), showed high validity to measure MVPA.

**Conclusions:**

This study was the first to examine the concurrent validity of low-cost trackers. Validity was strongest for the measurement of steps; there was evidence of validity for measurement of sleep in some trackers, and validity for measurement of MVPA time was weak throughout all devices. Validity ranged between devices, with Xiaomi having the highest validity for measurement of steps and VeryFit performing relatively strong across both sleep and steps domains. Low-cost trackers hold promise for monitoring and measurement of movement and sleep behaviors, both for consumers and researchers.

## Introduction

### Background

Physical activity (PA) and sleep are modifiable determinants of morbidity and mortality among adults and specifically contribute to the development of diseases such as obesity, type 2 diabetes, cardiovascular diseases, low quality of life, and mental health problems [1-6]. Engaging in at least 30 min of moderate-to-vigorous physical activity (MVPA) per day, getting between 7 and 9 hours of total sleep time (TST) per night, and spending relatively more time on light PA rather than being sedentary are associated with beneficial health outcomes [1-6]. A large proportion of adults do not meet the guidelines for one or more of these behaviors [6,7]. PA and sleep are, together with time spent on sedentary behavior (SB), codependent behaviors: they are part of one 24-hour day, and time spent on one behavior will impact the time spent on at least one of the other behaviors. It is, therefore, recommended to target these behaviors together [8].

Successful health promotion interventions rely on behavior change techniques that address modifiable determinants of health behavior [9]. A behavior change technique reported as both effective [10] and highly appreciated by users [11,12] is self-monitoring of health behavior. Self-monitoring refers to keeping a record of the behavior that is performed [13]. Self-monitoring tools provide opportunities for self-management of health as well as for remote activity tracking by health care providers as part of a patient’s treatment regimen [14]. Subjective ways of self-monitoring, such as self-report using retrospective measures (eg, diaries and questionnaires), often come with high participant burden and reporting biases [14]. Self-reported sleep duration in sleep logs showed an overestimation in comparison with objective measurements, especially when sleep duration was below the recommended health norms [15]. Activity trackers conversely offer automated, objective, and convenient means for self-monitoring PA and sleep. This paper focused on self-monitoring via consumer-based activity trackers as intervention tools for PA and sleep, more specifically by investigating the validity of low-cost trackers. Such trackers rarely monitor SB [16,17], which is why SB, although important in 24-hour movement behaviors, falls outside the scope of this paper.

Activity trackers may include pedometers, smartphone-based accelerometers, and accelerometers in advanced electronic wearable trackers or in smartwatches. However, pedometers do not provide information on sleep, and smartphone-based accelerometers have shown lower accuracy when measuring PA compared to advanced electronic wearable trackers [18], making advanced electronic wearable trackers and smartwatches more suitable to accurately self-monitor PA and sleep. Smartwatches (eg, Apple Watch) offer several other functions apart from activity tracking such as communication and entertainment and are usually more expensive than advanced electronic wearable trackers (eg, Fitbit Charge). Advanced electronic wearable trackers (termed as activity trackers hereafter) are usually wrist-or belt-worn, provide 24-hour self-monitoring, and often include real-time behavioral feedback or more detailed feedback shown after synchronization with other electronic devices (eg, tablet, smartphone, or PC) [19]. Several commercial activity trackers are available to the public and are increasingly integrated into effective intervention programs to improve activity behaviors [20,21].

There has been an increased interest by adults in activity trackers. For example, in Flanders, Belgium, 8% of adults owned an activity tracker (22% owned a type of wearable, including sports watches and smartwatches) in 2018 compared with only 2% (8% owned a type of wearable, including sports watches and smartwatches) owning one in 2015 [22]. Characteristics of activity trackers may impact their continued use and further adoption. Cost is likely to be a barrier to increased adoption of higher-end trackers [19,23]. Indeed, activity trackers appear to be used less among adults who are less educated, unemployed [19], and have a lower income [22]. Notably, unhealthy lifestyles such as insufficient PA [24] and insufficient sleep duration [25] are more prevalent among people of lower to medium socioeconomic status (SES) than among those of higher SES. Therefore, providing accurate, low-cost options to self-monitor PA and sleep in their daily lives is crucial for public health, as a lack of valid low-cost trackers may increase the health and digital divide between lower and higher income groups in the society. However, nonadoption of activity trackers in low SES populations can probably not only be attributed to the high cost of the devices but may also be a matter of priorities and affordances. Further research in this area is necessary. Having valid low-cost trackers not only plays a role in low SES populations but also in the general population; cost-effective solutions are needed for scaling up interventions in a public health context where financial resources are limited [26]. Having accurate, low-cost activity trackers can be expected to increase the feasibility of scaling up interventions that rely on activity trackers.

The unequal access to valid tools because of cost barriers is often studied within health literacy conceptual frameworks. Health literacy refers to having the ability and motivation to take responsibility for one’s own health [27]. Low health literacy has been associated with worse health outcomes [27], and improving access to tools that can help understand their own health behavior via self-monitoring and taking responsibility to take care of one’s own health may improve health literacy. There is increasing attention to expanding the health literacy model to electronic health (eHealth) literacy or digital health literacy, defined as the ability of people to use emerging technology tools to improve or enable health and health care [28]. Digital health literacy appears to be associated with a lower SES [29].

More specifically, the importance of accuracy of low-cost trackers can also be understood from the technology acceptance model that emphasizes the need for trust and perceived usefulness, together with perceived ease of use, of a tool before users are willing to adopt them [30].

When using activity trackers, their accuracy needs to be established to avoid counterproductive effects, such as falsely signaling that people are meeting guidelines and need not make any extra efforts, whereas, in fact, these people may not reach the sufficient sleep or PA levels [31]. Conversely, an underestimation of actual behavior can also cause people to get demotivated and to no longer make efforts to do better [32]. Accuracy of the tracker has also been cited by users as the trackers’ most important characteristic [19]. To effectively use wearable activity trackers for health self-management in daily life, accuracy needs to be assessed in free-living settings because laboratory-based validity studies tend to overestimate validity [18]. The validity to measure PA in free-living conditions has been examined for several activity trackers, such as Fitbit One, Zip, Ultra, Classic, Flex [16-18,33-38], Misfit Shine [18], and Withings Pulse [18]. In general, studies found the highest validity for Fitbit trackers [18]. Most validated trackers showed high correlations with an ActiGraph accelerometer for number of steps [16,39,40]. MVPA is less often studied and less accurately measured by activity trackers than step count [39]. Activity trackers showed moderate-to-strong correlations with ActiGraph accelerometers on MVPA, with Fitbit trackers and Withings Pulse showing the highest accuracy [39]. In addition, for TST, several wearable activity trackers currently on the market have been assessed for validity, including Fitbit (Flex and Charge HR) [41-43], Withings Pulse [39,41], Basis Health Tracker [41], Garmin [44], and Polar Loop [44]. Validity results for TST were very divergent, ranging from low to strong validity, with Fitbit again showing better validity [39,44]. The accuracy of PA and/or TST depends on the position where the tracker is worn, for example, the wrist vs the hip [33], and can be improved by combining accelerometry with the heart rate measurement [45,46].

The cost of the trackers in the abovementioned published validation studies was often not reported, but their price in the current market (at the end of January 2019) ranged from €50 (US $56) to €130 (US $146) for an unused, basic model (with Misfit Flash as the exception at €42 [US $47]). Most trackers that are popular in the consumer market and that are reported on in scientific publications cost more than €50 (US $56) and commonly more than €100 (US $112) [14]. A recent industry report states that when spending less than US $50, users are likely to get a product of mediocre accuracy [47], although it is unclear whether this statement was empirically based. To our knowledge, only 2 studies have examined the validity of low-cost trackers. Wahl et al’s [48] study of the Polar Loop (price in June 2019 around €60 [US $67]), Beurer AS80 (price in June 2019 around €42 [US $47]), and Xiaomi Mi Band (price in June 2019 around €25 [US $28]) suggested that only the Mi Band had good validity for step count. However, this study was conducted in a laboratory and not in free-living conditions. In one other study, the validity of the Xiaomi Mi Band for measuring TST was evaluated relative to a manual switch-to-sleep-mode measurement, with positive results [49]. However, this study did not use an objective measurement tool for comparison. We are not aware of any validation studies of low-cost activity trackers against objective measurement methods conducted in free-living conditions, and many of the most commonly available low-cost trackers do not appear to have been validated in any form.

In summary, wearable activity trackers can be a useful tool in health promotion and remote treatment monitoring for PA and TST. However, high costs and accuracy concerns may be barriers to widespread adoption [50]. Assessing the validity of low-cost trackers may play a major role at the population level to encourage health behavior in the future and among low SES groups who are most at risk for poor health and in need of healthy behavior promotion. To enable activity self-monitoring in daily life, the accuracy of low-cost wearable activity trackers needs to be established in free-living conditions. Current validation studies have mainly focused on wearable activity trackers that cost above €50 (US $56).

### Objectives

This study aimed to assess the validity of low-cost wearable activity trackers among adults (≤€50 [US $56]) for the objective measurement of PA and TST in daily life against free-living gold standards (ActiGraph GT3X+ accelerometer and BodyMedia SenseWear). This study was exploratory in nature and did not have firm hypotheses regarding the validity of specific low-cost trackers. However, it may be expected that trackers with heart rate monitoring are more accurate than those without heart rate monitoring. This may be because heart rate measurement contributes to a more accurate estimate of intensity and energy expenditure, resulting in a more accurate discrimination between activity and nonactivity [45,46].

## Methods

### Participants and Procedure

A concurrent validity study among adults was designed in which a low-cost tracker was validated against a free-living condition standard for steps, active minutes (MVPA), and TST. A high-cost tracker (Fitbit Charge 2) was also validated against these gold standards, to compare with validation outcomes for the low-cost trackers. In each participant, three 24-hour observation days were collected for each low-cost tracker. Power analyses (run in G*Power 3.1.9.2) suggested that to detect a 2-tailed significant correlation (H_1_) of 0.49 to 0.90, with 80% power (values based on the study by Brooke et al [44]), a sample size of between 6 and 29 was required.

A total of 20 healthy participants aged between 18 and 65 years living in Flanders, Belgium, were recruited using convenience sampling. Inclusion criteria were having no current physical limitations, medical conditions, or psychiatric conditions that may impact movement or sleep. Descriptive information collected on participants consisted of age, sex, self-reported height and weight, and highest attained education. All participants read and signed an informed consent form. The Ethics Committee of the University Hospital of Ghent approved the study protocol (B670201731732).

### Instruments

#### Convergent Measure

As this is a free-living study, the ActiGraph GT3X+ (ActiGraph) triaxial accelerometer was used as a reliable and valid reference for measuring step count [51-53] and MVPA [54,55]. The GT3X+ has been shown to be a valid measure of both step count compared with direct observation (percentage error <1.5% [52]; percentage error ≤1.1% [53]; and intraclass correlation coefficient [ICC]≥0.84 [51]) and MVPA compared with indirect calorimetry (*r*=0.88) [54]. Accelerometer data were initialized, downloaded, and processed using ActiLife version 5.5.5 software (ActiGraph). The Freedson Adult cut-points were applied to categorize PA measured by using the ActiGraph accelerometer (sedentary activity=0-99 counts per min, light activity=100-1951 counts per min, moderate activity=1952-5723 counts per min, and vigorous activity ≥5724 counts per min) [54]. A 15-second epoch was used when downloading the data. The ActiGraph GT3X+ was fitted to the right side of the participants’ waist in accordance with the manufacturer’s instructions. Only days with valid data of the ActiGraph were included in the analysis. A valid day was defined as a 24-hour period in which at least 10 hours of data wear time were recorded [56]. Nonwear time was analyzed as a run of zero counts lasting more than 60 min with an allowance of 2 min of interruptions. Using this algorithm, the risk of misclassification of nonwear time as sedentary time was avoided [57].

The BodyMedia SenseWear (BodyMedia Inc) is a portable multisensor device that can provide information regarding the total energy expenditure, TST, circadian rhythm, and other activity metrics. In this study, the SenseWear was used as the reference for sleep duration. SenseWear has been validated as a measure of TST compared with polysomnography (*r*=0.83; SE of estimate 37.71) [58]. Data were analyzed in SenseWear Professional 8.1 software [59]. The SenseWear was placed over the triceps muscle on the nondominant arm between the acromion and olecranon processes, in accordance with the manufacturer’s instructions.

#### Low-Cost Activity Trackers

**Figure 1:**
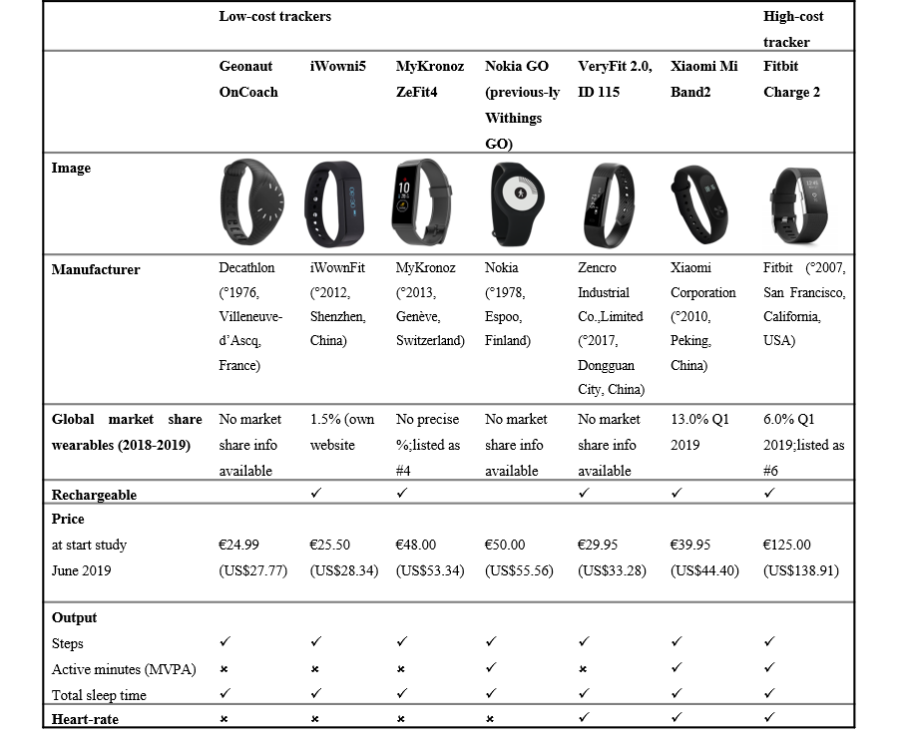
Tracker characteristics.

In total, 6 low-cost activity trackers were selected (Figure 1) based on their price at the time of the study (≤€50 [US $56]), their market share (eg, MyKronoz and Xiaomi), whether or not they included a heart rate measurement and output (steps, MVPA or active minutes, and TST), and availability from popular web-based purchase sites in Europe where the study was conducted. Furthermore, we tested the Fitbit Charge 2 to also include a comparison between a low-cost activity tracker and a validated high-cost activity tracker. Fitbit was selected as a high-cost activity tracker because it was one of the most popular activity trackers on the market at the start of the study and was already validated for measuring steps, MVPA, and TST [17]. All participants received a Wiko smartphone in loan (Lenny 3, Android 6.0 Marshmallow, price €99.99 [US $119.80] in June 2019) to pair the trackers with, to cancel out any potential individual differences in smartphone pairing.

All devices measured steps and TST. Only Xiaomi, Nokia, and also Fitbit used a specific variable that quantifies intensive forms of PA. These 3 devices reported *active minutes* with no further subdivision. As all the devices set a goal of 30 min PA per day (similar to the MVPA recommendations for adults), it was assumed that the measured variable corresponded to MVPA as measured by the ActiGraph. However, specific information regarding intensity cut-points is not publicly available. TST was used, excluding daytime naps, for comparison with the SenseWear that was only worn at night. Only Fitbit, VeryFit, and Xiaomi measured the heart rate. Data were extracted using the proprietary software for all devices, in the same fashion that a consumer would use the software, and were visually checked for outliers.

### Free-Living Protocol

**Figure 2:**
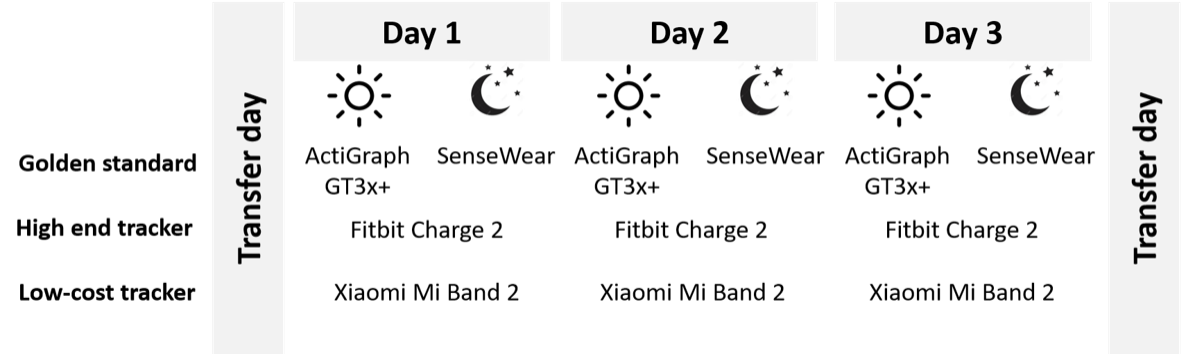
An example of the measurement protocol for one period.

As it was not feasible or comfortable to wear all trackers at the same time, participants were instructed to wear one of the low-cost devices in combination with the Fitbit tracker on their nondominant wrist. They were also instructed to simultaneously wear the ActiGraph on their hip during daytime and the SenseWear on their upper arm at nighttime. Furthermore, the participants were provided with a diary to write down the time they put on and took off the devices. This way it could be checked that the devices were always worn simultaneously. If this was not the case, data of the device that was worn separately were deleted to avoid a mismatch of the measurements. Participants received the 6 low-cost trackers in a random order. The position of the low-cost and high-cost tracker on the nondominant wrist (first or second in distance from the wrist) was varied across days. Each tracker was worn for a period of 3 consecutive days and nights. A period of 3 days and 3 nights was chosen to balance between achieving sufficient data for the question under study without burdening the participants. Between 2 periods, a 1-day gap allowed for switching the devices. During daytime, the devices were worn during all waking hours, except during water-based activities. When participants went to bed, they were asked to remove the ActiGraph and put on the SenseWear instead. In Figure 2, a typical measurement period for one device is shown.

PA or TST may differ between weekdays and weekend days. Although this study did not intend to explain differences in PA or TST but rather the degree of agreement between 2 measurements on any given day, a difference in how often a tracker was measured on a certain day rather than another day may influence validity results. For example, validity has shown to be lower for measuring a low number of steps or high number of steps. Our study design controlled for this potential influence by randomly varying the days across participants on which a particular tracker was worn. Across all data points, we would then expect all measurement days to be relatively equally represented, as was the case in our study. The percentage of weekend days in total measurement days ranged between 25% and 33%. In addition, on particular weekdays, there were very few differences (2%-9% difference between the tracker with the lowest number of measurements on a certain day and the tracker with the highest number of measurements on a particular day).

### Statistical Analysis

Analyses were performed using IBM SPSS Statistics version 24.0 (SPSS Inc). All analyses were performed on a daily measurement level, counting a measured day as a unit of analysis. Analyses consisted of measures of agreement, systematic differences, and bias and limits of agreement. Measures of agreement (equivalence testing) included the Spearman correlation coefficient (*r*) to examine the association between steps, active minutes, and TST measured by trackers and convergent measure (also illustrated in scatter plots). As sleep and PA data were nonnormally distributed, (1) a Spearman correlation, a nonparametric statistical test, was used instead of a Pearson correlation and (2) *ICC* (absolute agreement, 2-way random, single measures, 95% CI) that reflects the effect of individual differences on observed measures. Measures of systematic differences included mean absolute percentage errors (MAPEs) of tracker measurements compared with those of the convergent measure. MAPEs were calculated with the following formula: mean difference activity tracker−convergent measure × 100/mean gold standard. Bland-Altman plots with their associated limits of agreement were used to examine biases between measurements from the trackers and the convergent measure. The following cutoff values were used to interpret the Spearman correlation coefficient: <0.20=very weak, 0.20 to 0.39=weak, 0.40 to 0.59=moderate, 0.60 to 0.79=strong, and 0.80 to 1.0=very strong [60]. The cutoff values to interpret the ICC were as follows: <0.60=low, 0.60 to 0.75=moderate, 0.75 to 0.90=good, and >0.90=excellent [48].

A series of linear mixed effects models with restricted maximum likelihood estimation were used to examine the association between steps, MVPA minutes, and TST measured by the commercial trackers and convergent measures, accounting for the structure of the data (repeated measures clustered within participants). The pattern of results was similar to that obtained from the abovementioned analyses. Data are, therefore, presented in Multimedia Appendix 1.

## Results

### Descriptive Statistics

A total of 3 participants discontinued their participation in the study: one participant dropped out at the start of the study because of the combination of high perceived burden of the research protocol and a busy personal schedule, and consequently, no data were collected and analyzed from this participant; one participant was not able to meet the protocol toward the end of the study because of conflict with his/her work schedule; and one participant had to end participation because of an unexpected hospital admission (17/20, 85% retention rate). The average age of the analyzed sample of participants who started the study (n=19) was 37.6 (SD 13.4) years; of 19 participants, 13 were female. The sample was highly educated, with 17 participants having achieved a higher education degree (academic or nonacademic). Their average BMI was 23.5 (SD 4.4) kg/m^2^. Two participants were overweight (BMI of 25-30 kg/m^2^), and 2 participants were obese (BMI ≥30 kg/m^2^). The level of MVPA measured at baseline with the International Physical Activity Questionnaire varied from 10 to 351 min per day (SD 91) [61,62].

All participants owned a smartphone; 5 of 19 participants had previous experience with wearable trackers (n=3; Fitbit). As can be expected in a highly educated sample, they were all very familiar with digital tools and required little assistance in installation or usage. We did not expect any impact of participants’ experience on the validity measurements, as (1) these would not have a differential effect of any potential misuse between different trackers and (2) control procedures were put in place to prevent any misuse. Potential misuse could consist of a wrong placement of the tracker. Participants received a thorough briefing at the start of the study and a daily check-up of any issues to ensure any baseline differences in familiarity with digital tools were canceled out and to reduce the risk for misuse. No issues with misuse were noted.

### Issue of Usability With Low-Cost Trackers

In total, each device was intended to be tested for 60 days. As one of the participants did not start, the maximum number of potential measurement days per tracker was reduced to 57. The number of days of available data varied per tracker because of dropouts at the end of the study by some participants and because of technical issues experienced with some trackers, which resulted in fewer days of available data.

Of 57 measurement days, VeryFit had 55 (96%) measured days for PA (lost days: 2 because of no data shown in the app) and 51 (86%) measured days for sleep (lost days: 3 because of participant noncompliance and 3 because of no data shown in the app). Of 57 measurement days, iWown had 52 (89%) measured days for PA (lost days: 4 because the tracker did not pair and 1 because of no data shown in the app) and 51 (89%) measured days for sleep (lost days: 4 because the tracker did not pair and 2 because of no data shown in the app). Xiaomi was not worn by 2 participants because of dropping out, reducing potential measurement days to 51. Of 51 measured days, Xiaomi had 48 (94%) measured days for PA (lost days: 2 because of participant noncompliance and 1 because of no data shown in the app) and 44 (86%) measured days for sleep (lost days: 6 because of participant noncompliance and 1 because of no data shown in the app). Of 57 measured days, Nokia had 49 (86%) measured days for PA (8 lost days because of no data shown in the app) and 46 (81%) measured days for sleep (lost days: 8 because of no data shown in the app and 3 because of participant noncompliance). MyKronoz was not worn by 3 participants because of dropping out; one participant accidentally removed the data, reducing potential measurement days to 45. Of 45 measured days, MyKronoz had 40 (89%) measurement days for PA (5 lost days because of no data shown in the app) and 24 (53%) for sleep (lost days: 11 because of no data shown in the app and 10 because of participant noncompliance). Of 57 measured days, Geonaut had 37 (65%) measured days for PA (lost days: 12 because of no data shown in the app, 9 because of the device not pairing, and 5 because of participant noncompliance) and 30 (53%) measured days for sleep (lost days: 9 days because of the device not pairing, 8 because of no data shown in the app, and 4 because of participant noncompliance).

Participants were especially frustrated about a device not pairing, as this meant they had to reinstall the tracker and also lost their past activity history. Thus, VeryFit and Xiaomi showed little data loss because of usability problems, whereas especially for Geonaut and MyKronoz, data were lost because of usability problems. In general, more data were lost for sleep than for PA. Usable data in the analyses were further reduced because of technical issues experienced with the convergent measures, which resulted in fewer days of data for which comparisons could be made (the number of usable data points are shown in all tables).

### Validity of Low-Cost Trackers

#### Physical Activity

Table 1 shows the mean steps, mean minutes of MVPA, and the corresponding standard deviations for all trackers for measuring steps and MVPA.

**Table 1:** Mean steps and minutes of moderate-to-vigorous physical activity per day measured by the low-cost trackers, Fitbit and ActiGraph.

Tracker		Number of measured days	Mean (SD)	Range
**Number of steps per day**				
	Geonaut	37	8026 (4352)	657-19,413
	iWown	51	7668 (5169)	259-22,759
	MyKronoz	40	10,431 (4764)	485-24,493
	Nokia	50	5896 (3113)	325-13,976
	VeryFit	55	7320 (4481)	649-22,628
	Xiaomi	48	7317 (4535)	369-20,866
	Fitbit	307	9662 (4866)	451-24,664
	ActiGraph	316	8126 (4314)	188-23,121
**Number of minutes of** **moderate-to-vigorous physical activity** **per day**				
	Nokia	49	5 (12)	0-52
	Xiaomi	46	80 (48)	0-190
	Fitbit	305	45 (49)	0-239
	ActiGraph	328	41 (31)	0-150

Agreement testing for steps diverged between the Spearman *r* coefficient and ICC (Table 2). All trackers, except iWown, showed strong (Nokia, Geonaut, VeryFit, and MyKronoz) to very strong (Xiaomi and Fitbit) agreement with the ActiGraph measurements based on the Spearman *r* coefficient (all above 0.60). On the basis of ICC, MyKronoz, iWown, and Nokia showed low agreement (ICC<0.60), whereas Geonaut had moderate and Xiaomi, Fitbit, and VeryFit had a good agreement with the ActiGraph measurements (ICC=0.75-0.90). These coefficients are in line with the interpretation of the MAPE scores, showing the largest mean deviation from the ActiGraph measurements for iWown (35.28%) and the smallest for the Xiaomi tracker (17.14%).

For measuring MVPA, correlations between the MVPA measurements of the trackers and the ActiGraph accelerometer were weak for Nokia and Xiaomi and moderate for Fitbit (Table 2). The ICC showed low agreement for MVPA between all 3 trackers and the ActiGraph accelerometer (ICC<0.60). The MAPE scores also indicate very large mean deviations from the ActiGraph measurements for MVPA (>100%), which confirm the low accuracy of the trackers for measuring MVPA.

**Table 2:** Correlation coefficients, intraclass correlation coefficients, associated 95% CI of the measurements, and mean absolute percentage error scores for measuring steps and moderate-to-vigorous physical activity.

Tracker		n	Spearman *r* (95% CI)	Intraclass correlation coefficient	95% CI	Mean absolute percentage error (%)
**Steps**						
	Geonaut	36	0.63^a^ (0.31 to 0.87)	0.68^a^	0.46 to 0.82	24.63
	iWown	50	0.53^a^ (0.16 to 0.77)	0.51^a^	0.28 to 0.69	35.28
	MyKronoz	38	0.77^a^ (0.45 to 0.95)	0.59^a^	0.22 to 0.79	25.79
	Nokia	50	0.77^a^ (0.51 to 0.94)	0.56^a^	0.27 to 0.74	22.62
	VeryFit	54	0.78^a^ (0.61 to 0.89)	0.82^a^	0.62 to 0.91	24.87
	Xiaomi	45	0.91^a^ (0.81 to 0.97)	0.90^a^	0.77 to 0.95	17.14
	Fitbit	300	0.91^a^ (0.86 to 0.94)	0.87^a^	0.66 to 0.93	25.73
**Moderate-to-vigorous physical activity**						
	Nokia	16	0.24 (−0.11 to 0.50)	0.18	−0.10 to 0.44	108.17
	Xiaomi	45	0.26 (−0.08 to 0.54)	0.15	−0.08 to 0.39	293.29
	Fitbit	298	0.56^a^ (0.47 to 0.63)	0.56^a^	0.48 to 0.64	114.30

^a^*P*<.001.

**Figure 3:**
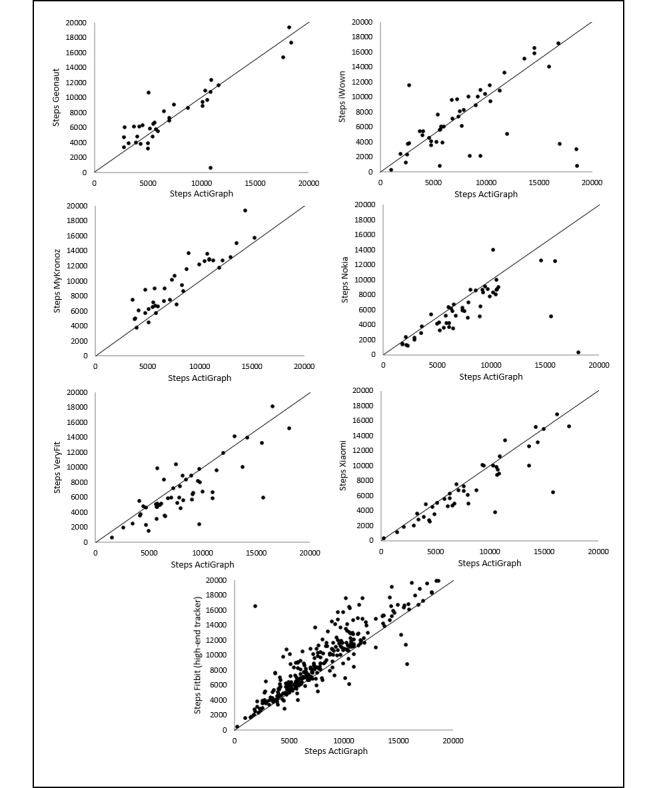
Correlations between steps estimates per day from the trackers and the ActiGraph.

**Figure 4:**
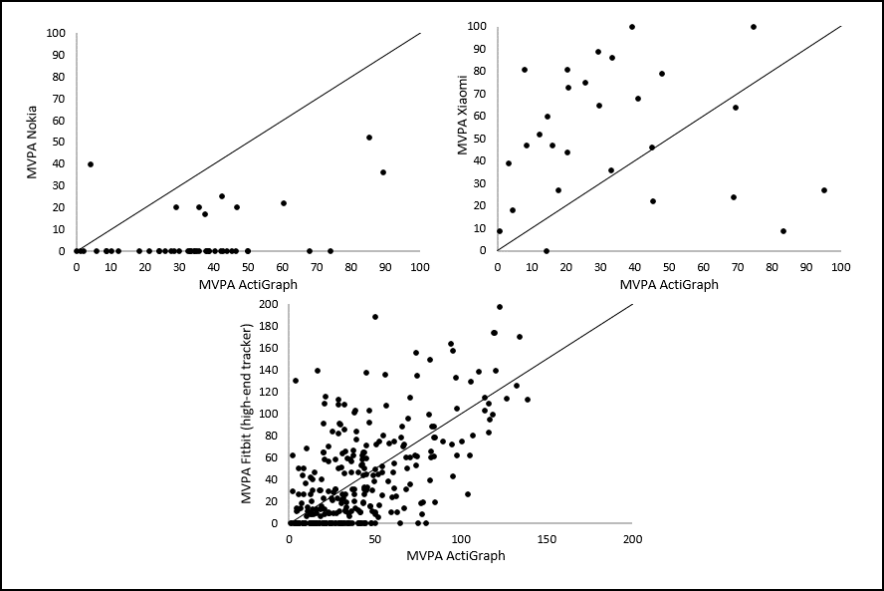
Correlations between moderate-to-vigorous physical activity estimates per day from the trackers and the ActiGraph.

Correlations for steps and MVPA are illustrated in Figures 3 and 4. Scatter and deviation of the points around the line that reflects the perfect agreement between the measurements are larger for measuring MVPA than for measuring steps. The largest scatter for measuring steps is found for iWown (Figure 3). On the basis of scatterplots, a careful statement on overestimation or underestimation of the measurement of the trackers can be made. This is based on the location of the data points relative to the line that represents the perfect agreement between the measurements. For Xiaomi, Nokia, and VeryFit, the majority of the data points are located below that line, meaning an underestimation of the number of steps. For iWown, MyKronoz, and Fitbit, the majority of the data points are located above the line, meaning an overestimation of the number of steps. For Geonaut, no clear underestimation or overestimation is visualized. A large scatter for all 3 trackers that measure MVPA was observed, with no obvious relation between the MVPA measurements of the trackers and the MVPA measurements of the ActiGraph. For Nokia, an underestimation is visualized, and for Xiaomi, however, an overestimation is visualized. For Fitbit, no clear underestimation or overestimation is visualized.

**Figure 5:**
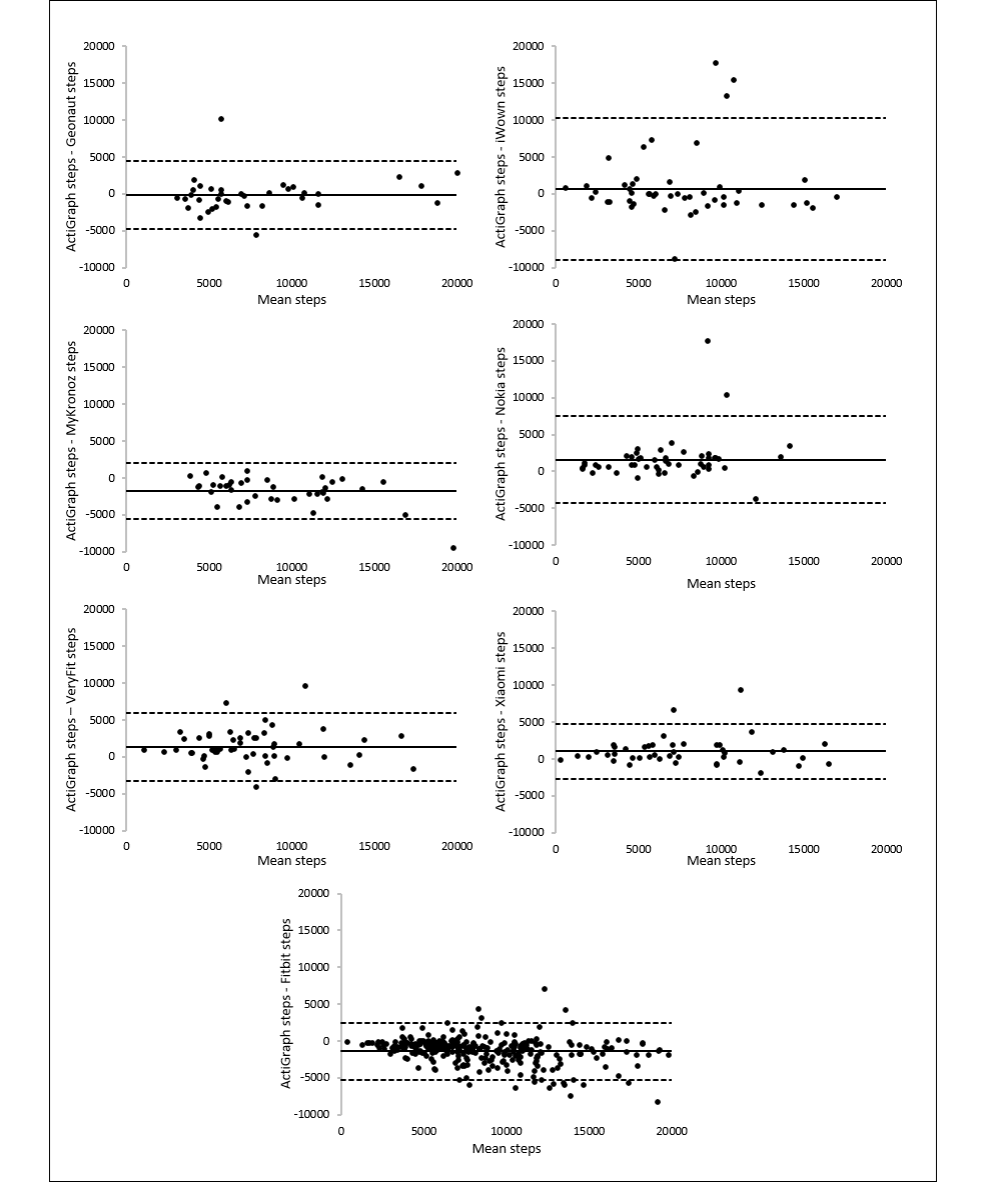
Bland-Altman plots of the trackers for measuring steps. The middle line shows the mean difference (positive values indicate an underestimation of the wearable and negative values indicate an overestimation) between the measurements of steps of the wearables and the ActiGraph and the dashed lines indicate the limits of agreement (1.96×SD of the difference scores).

**Figure 6:**
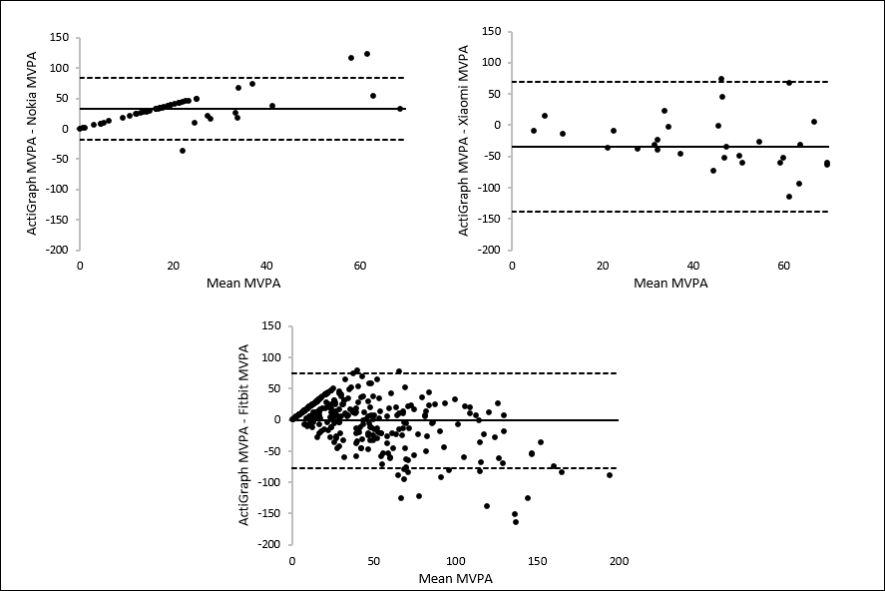
Bland-Altman plots of the trackers for measuring moderate-to-vigorous physical activity. The middle line shows the mean difference (positive values indicate an underestimation of the wearable, and negative values indicate an overestimation) between the measurements of moderate-to-vigorous physical activity of the wearables and the ActiGraph, and the dashed lines indicate the limits of agreement (1.96×SD of the difference scores).

These findings are also visualized by using Bland-Altman plots. Bland-Altman plots were used to visualize the differences between the steps and MVPA measurements of the ActiGraph accelerometer and each tracker (y-axis) against the average number of steps or number of minutes of MVPA of the measurements of these 2 devices (x-axis). Mean differences with the ActiGraph accelerometer and the limits of agreement are presented in Table 3 (illustrated in Figures 5 and 6 for steps and MVPA, respectively). A positive value of the mean difference indicates an underestimation of the measurements of the tracker compared with the ActiGraph measurements, whereas a negative value indicates an overestimation. The systematic overestimation or underestimation (mean differences) and the range between the upper and lower limits of the agreement reflect the accuracy of the measurements of the tracker compared with the measurements of the ActiGraph accelerometer. The broader the range between the lower and the upper limit, the less accurate the measurements are.

For measuring steps and MVPA, the table and the plots (Figures 5 and 6) all showed large limits. The Xiaomi tracker showed the narrowest limits (7450 steps) for measuring steps, whereas iWown showed the broadest limits (19,263 steps). These results are in line with the interpretations of validity findings based on the Spearman *r*, the ICC, and the MAPE score.

For MVPA, the ranges between the lower and upper limit of agreement are very large, indicating a low accurate measurement by all 3 trackers measuring MVPA. The Bland-Altman plots showed the broadest limits for Xiaomi (207.64 min) and the narrowest limits for Nokia (101.80 min).

Thus, several but not all low-cost trackers showed high accuracy to measure steps. Xiaomi trackers even outperformed the Fitbit tracker in measuring steps. However, none of the trackers showed good accuracy to measure MVPA, including Fitbit, which did nevertheless reach a slightly higher validity than the low-cost trackers in measuring MVPA.

**Table 3:** Mean differences of activity measures with the ActiGraph accelerometer and limits of agreement of the activity trackers.

Tracker		n	Mean difference of steps (ActiGraph−Tracker)	Limits of agreement, range	Width of the limits of agreement
**Steps**					
	Geonaut	36	−146	−4802 to 4509	9311
	iWown	50	638	−8993 to 10,270	19,263
	MyKronoz	38	−1798	−5563 to 1967	7530
	Nokia	50	1609	−4229 to 7447	11,676
	VeryFit	54	1356	−3276 to 5989	9265
	Xiaomi	45	1011	−2713 to 4737	7450
	Fitbit	300	−1369	−5238 to 2499	7737
**Moderate-to-vigorous physical activity**					
	Nokia	16	32.55	−18.35 to 83.45	101.80
	Xiaomi	45	−35.14	−138.96 to 68.68	207.64
	Fitbit	298	−1.27	−77.07 to 74.52	151.59

#### Total Sleep Time

Table 4 reports the mean minutes of TST and corresponding standard deviations for all trackers.

Spearman correlations between the TST measurements of the trackers and the TST measurements of the SenseWear armband show large diversity between trackers, ranging from very weak (Geonaut) to strong (VeryFit). The ICCs, however, indicate low agreement (ICC<0.60) between the measurements of all trackers and the measurements of the SenseWear. This could reflect a systematic underestimation or overestimation of TST by the trackers, which is not evident from the Spearman *r* coefficient. The MAPE scores of all trackers also indicate a large mean deviation from the SenseWear measurements for TST, ranging from 20.57% for Fitbit to 39.08% for Xiaomi. The correlation coefficients, ICC values, associated 95% CI, and MAPE scores for measuring TST are shown in Table 5.

**Table 4:** Mean total sleep time per day measured by the low-cost trackers, Fitbit and SenseWear.

Tracker		Number of measured days	Total sleep time (minutes), mean (SD)	Range (minutes)
**Total sleep time**				
	Geonaut	30	341 (123)	110-589
	iWown	52	421 (108)	91-624
	MyKronoz	24	457 (143)	70-746
	Nokia	46	464 (108)	247-743
	VeryFit	51	472 (59)	193-614
	Xiaomi	44	495 (87)	285-695
	Fitbit	287	414 (91)	68-733
	SenseWear	147	373 (83)	112-653

**Table 5:** Correlation coefficients, intraclass correlation coefficients, associated 95% CI of the measurements, and mean absolute percentage error scores for measuring total sleep time.

Tracker		n	Spearman *r* (95% CI)	Intraclass correlation coefficient	95% CI	Mean absolute percentage error (%)
**Total sleep time**						
	Geonaut	15	0.04 (−0.45 to 0.60)	0.05	−0.44 to 0.52	26.59
	iWown	24	0.57^a^ (0.19 to 0.84)	0.52^a^	0.18 to 0.76	21.33
	MyKronoz	14	0.45 (−0.22 to 0.86)	0.40^a^	−0.07 to 0.74	38.15
	Nokia	14	0.66^b^ (0.30 to 0.88)	0.30^a^	−0.10 to 0.63	38.63
	VeryFit	24	0.73^b^ (0.48 to 0.83)	0.26	−0.11 to 0.61	30.73
	Xiaomi	21	0.21 (−0.34 to 0.68)	0.13	−0.13 to 0.45	39.08
	Fitbit	134	0.57^b^ (0.40 to 0.69)	0.46^b^	0.28 to 0.60	20.57

^a^*P*<.05.

^b^*P*<.001.

**Figure 7:**
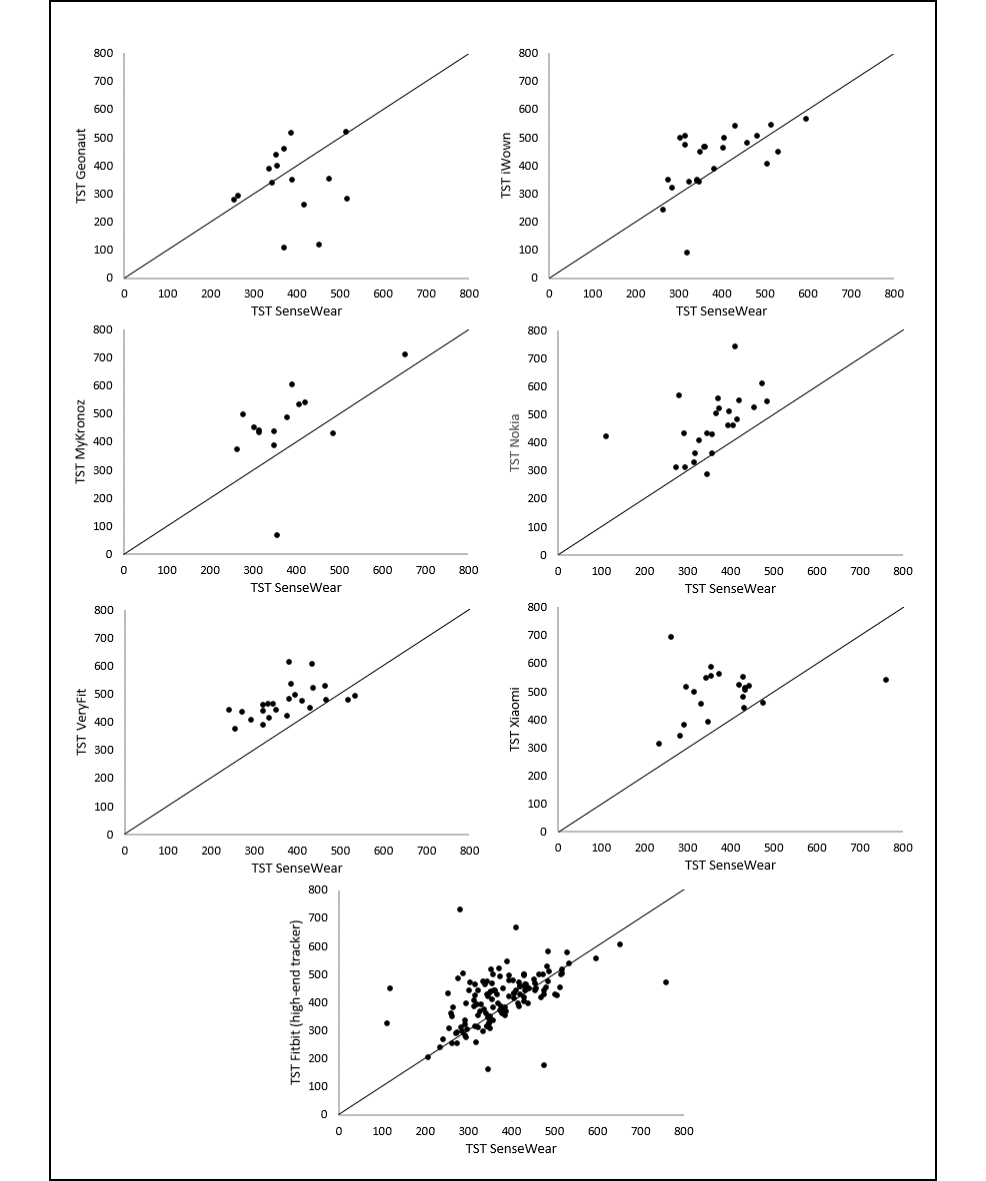
Correlations between total sleep time estimates from the trackers and the SenseWear.

The correlations for TST are also illustrated in Figure 7. This figure visualizes the large discrepancy between the Spearman correlation coefficient and the ICC, specifically evident for Nokia and VeryFit. Although a clear relation is visible between the measurements (Spearman *r*), almost all data points are above the line that represents the perfect agreement between the measurements. This indicates a systematic overestimation of the TST measurements of Nokia and VeryFit compared with the convergent measure. Figure 7 also shows the largest scatter for MyKronoz.

**Figure 8:**
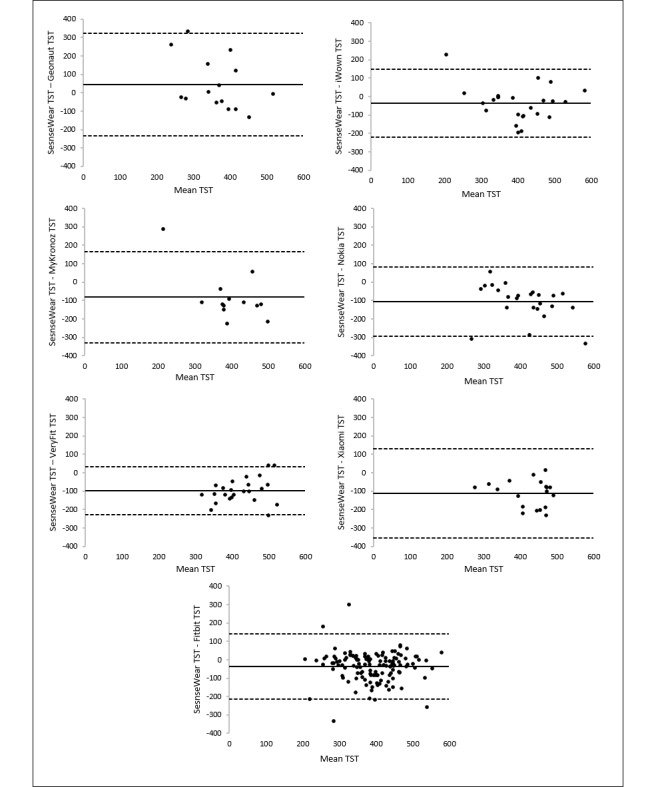
Bland-Altman plots of the trackers for measuring total sleep time. The middle line shows the mean difference (positive values indicate an underestimation of the wearable and negative values indicate an overestimation) between the measurements of total sleep time of the wearables and the SenseWear, and the dashed lines indicate the limits of agreement (1.96×SD of the difference scores).

Bland-Altman plots for TST revealed the smallest limits for VeryFit (263.39 min) and the broadest limits for Geonaut (558.25 min). These results are in line with the findings based on the Spearman *r* coefficient and the scatter of the data points. The mean differences with the SenseWear armband measurements and the limits of agreement are presented in Table 6 and illustrated in Figure 8.

**Table 6:** Mean differences of total sleep time measures with the SenseWear and limits of agreement of the activity trackers.

Tracker	n	Mean difference of total sleep time (SenseWear−smartwatch)	Limits of agreement, range	Width of the limits of agreement (minutes)
Geonaut	15	44.93	−234.19 to 324.06	558.25
iWown	24	−36.79	−221.22 to 147.63	368.85
MyKronoz	14	−82.29	−330.55 to 165.98	496.53
Nokia	24	−106.46	−293.72 to 80.80	374.52
VeryFit	24	−97.63	−229.32 to 34.07	263.39
Xiaomi	21	−112.14	−355.40 to 131.12	486.52
Fitbit	134	−36.91	−213.9 to 140.16	354.14

Thus, low-cost trackers showed low (eg, Geonaut and Xiaomi) to strong (eg, VeryFit) correlations to measure TST, with some trackers such as VeryFit and Nokia systematically overestimating TST. Fitbit showed low (based on ICC) to moderate (based on the Spearman *r* coefficient) validity to measure TST and was outperformed by VeryFit to measure TST on all indicators of accuracy.

## Discussion

### Principal Findings

This study examined the validity of low-cost trackers (≤€50 [US $56]) for measuring adults’ steps, moderate-to-vigorous PA, and TST in free-living conditions. In general, the low-cost trackers were most accurate in the measurement of steps, somewhat accurate for the measurement of sleep, and lacked validity for the measurement of MVPA time. Validity ranged widely between the various low-cost trackers tested. The performance of the best of the low-cost trackers approached or even exceeded that of the Fitbit Charge 2 (the high-cost comparison tracker), whereas the worst of the low-cost trackers had weak validity. Notably, VeryFit 2.0 performed relatively strongly across both sleep and steps domains, whereas the Xiaomi Mi Band 2 appeared to have the highest validity for the measurement of steps.

The finding that many of the low-cost trackers are accurate for measuring steps is promising, given that steps is the metric reported by users of trackers as being of most interest [63]. We found that the low-cost trackers were most accurate for measuring steps in comparison with sleep and minutes of MVPA. This order for validity (ie, measuring steps more accurately than measuring sleep and in turn more accurately than measuring MVPA) is consistent with findings for these metrics in high-cost trackers [39], although in our study, the low-cost trackers demonstrated weak-to-moderate validity for MVPA minutes (Spearman *r* ranged from 0.24 to 0.56), whereas previous research in high-cost trackers has suggested moderate-to-strong validity (eg, Ferguson et al’s [39] study of high-cost trackers reported Pearson *r* ranging from 0.52 to 0.91). It is possible that some of the differences between the reference values for MVPA derived from the ActiGraph accelerometers and the values recorded by the low-cost trackers may have originated from a measurement error associated with the reference device. Furthermore, a possible explanation for the weak-to-nil validity found in our study could be that the PA variables measured by the low-cost trackers were not explicitly identified as MVPA. However, because all devices had set a goal of 30-min PA per day (similar to the MVPA recommendations for adults), we assumed that the measured variable corresponded to MVPA as measured by the ActiGraph accelerometer. Nevertheless, specific information regarding algorithm intensity cut-points was not provided and publicly available from these low-cost trackers. Therefore, the discrepancies in this study may be a result of both definitional and measurement problems (eg, sensitivity algorithm). In this regard, it may be very useful in the future, when manufacturers provide more insight into the cut-points and algorithms that were used to translate the raw data into useful information (such as steps and minutes of MVPA).

Although research-grade accelerometers are the closest we have to a *gold standard* for the measurement of MVPA in free-living conditions, the MVPA values derived from them can vary by the order of magnitude depending on parameters such as epoch length and cut-points [64]. Furthermore, wear position has an impact on the validity of MVPA. Studies comparing the validity of research-grade accelerometers at different body locations consistently show that the hip position is more accurate than the wrist [65]. Despite the recognized superior validity of hip-worn accelerometers and trackers, over the past 5 years or so, there has been a shift for both consumer trackers and research-grade accelerometers to increasingly be designed for wrist wear, presumably because of improved logistics, such as comfort and convenience. This clear shift in the market highlights that validity should not be considered the be-all and end-all. Issues such as usability, compliance, and adherence are also important, although they tend to receive less attention in the scientific literature.

Evidence for the validity of the low-cost trackers for the measurement of sleep duration was mixed. Some trackers performed quite strongly. For example, the top performing tracker, VeryFit 2.0, demonstrated a Spearman *r* of 0.73 for TST compared with the reference device (SenseWear), which was actually superior to the Fitbit Charge HR (*r*=0.57). However, the Bland-Altman analyses revealed that VeryFit 2.0 tended to overestimate sleep by around 1.5 hours per night compared with the reference device. If this overestimation was consistent, it could be argued that the data might still be useful for self-monitoring changes in sleep over time. However, the Bland-Altman 95% limits of agreement spanned a range of 263 min, suggesting that the extent of overestimation varied considerably on different administrations. It, therefore, seems questionable whether the sleep estimates derived from VeryFit 2.0 are accurate enough to help a user meaningfully monitor/change their sleeping patterns.

The finding that low-cost trackers have strong validity for measuring steps and some validity for measuring sleep is likely to be of interest to public health researchers and clinicians alike. There is considerable interest in using activity trackers to intervene on lifestyle activities, with a recent meta-analysis finding positive evidence for short-term effectiveness but less evidence for sustained effects [66]. There is well-recognized usage attrition associated with activity trackers over time. For example, a 2017 study gave entry-level Fitbit trackers to 711 users and found that approximately 50% of participants had stopped using them at 6 months and 80% had stopped by 10 months [67]. The most common reasons for not using Fitbit was technical failure or difficulty (57%), losing the device (13%), or forgetting to wear it (13%). Nonetheless, low-cost devices fill an important gap in the consumer market between the high-cost activity trackers that are prohibitively expensive to provide to clinical or research cohorts at scale (unless sizable funding is available) but likely to be more aesthetically pleasing and acceptable to wearers than traditional pedometers [19,63]. The findings of this study, which highlight the Xiaomi Mi Band 2 and VeryFit 2.0 devices as having acceptable validity, are therefore helpful. We bought the trackers as individual buyers on the consumer market. Researchers intending to use these in large-scale research cohorts may purchase these at an even lower cost in bulk. Another promising feature of VeryFit 2.0 is that it has an application programming interface (API) that allows software developers to create custom software that can be integrated directly with the tracker (ie, data from the tracker can be sent automatically to the custom software). There is a growing trend for eHealth and mobile health research to use Fitbit and Garmin API [68-70]. Therefore, validated low-cost trackers with APIs offer new data collection and intervention possibilities.

Our study included trackers with and without heart rate measurements. All trackers with the highest validity included heart rate measures, whereas those without showed lower validity. However, we cannot conclude from this study that the heart rate function increased validity. Studies testing the same model with and without the heart rate function and assessing the validity of the heart rate measurement in itself would be needed to make this claim. The price of included trackers ranged from around €25 (US $28) to approximately €50 (US $55). The prices of the most accurate types, VeryFit 2.0 and Xiaomi Mi Band 2, are situated in the middle of this range (€30-€40 [US $33-US $44]). This yields two models that are very attractive and accessible to the general public. Thus, price may not be the determining factor in the validity of the trackers: more expensive within this range is not necessarily better. On the contrary, we cannot conclude that price plays no role and that trackers even less expensive than those included here (<€25 [US $28]) may also be valid. Indeed, a study on pedometers provided for free as gadgets with cereal boxes found that those were not valid [71].

Although validity evidence from this study for low-cost devices measuring steps, MVPA, and TST is not unequivocally good across the devices, user experience is also extremely important. A device that has high validity may not necessarily have a positive user experience. Future research examining the user experience of low-cost trackers (eg, focusing on issues such as functionality, reliability, and ease of use, both of the device itself and its accompanying app) will be valuable. Our preliminary experiences suggest that the user experience of the low-cost trackers may be less positive than that of the high-cost trackers (eg, we tended to experience fewer technical issues with Fitbit trackers than with the other devices in this study). It can be assumed that the higher price of the high-cost trackers is partly determined by the investments made by the manufacturer to improve the user experience and to better develop the app supporting the tracker. Moreover, the low-cost activity trackers appear most valid for measuring steps. Pedometers that count steps are available at an even lower cost, but unlike activity trackers, they offer little additional functionality (eg, feedback, information, and social support) in an accompanying app and are considered less usable by people than activity trackers [72-74]. Further work to explore these issues more rigorously and in greater depth is warranted.

### Strengths and Limitations

A strength of our study is that it is the first to scrutinize the validity of low-cost trackers addressing an important gap in the scientific literature to date. Methodological strengths of the study are the relatively large number of devices that were tested using the same methodology (allowing a direct comparison of the devices’ performance), the devices that tested multiple metrics (steps, MVPA, and sleep), and that efforts were made to minimize bias, for example, by randomizing the order in which participants wore the devices. Limitations included that our sample was relatively young and healthy. On the basis of previous literature, it seems likely that validity for measuring steps is likely to be somewhat lower in older and clinical populations (eg, obese) [75]. As already noted, our reference devices were research-grade accelerometers with known validity limitations of their own. Therefore, they represent convergent validity rather than criterion validity, and there is a risk that we may be underestimating the low-cost trackers’ true validity. A further limitation is that this is a fast-moving field with new devices continually entering and exiting the market. In particular, since our study started, the Xiaomi MiBand 2 is replaced by its successor, the MiBand 3. Therefore, it would be beneficial that future research continuously investigates the validity of new low-cost trackers and other emerging devices. Furthermore, having an insight into the used algorithms and used cutoffs would be beneficial.

### Conclusions

This study was the first to examine the validity of low-cost trackers. It found that validity was strongest for the measurement of steps; there was some evidence of validity for the measurement of sleep, whereas validity for the measurement of MVPA time was weak. Validity ranged between devices, with Xiaomi having the highest validity for the measurement of steps and VeryFit performing relatively strongly across both sleep and steps domains. The tested low-cost trackers hold promise for the cost-efficient measurement of movement behaviors. Further research investigating the user experience of low-cost devices and their accompanying apps is needed before these devices can be confidently recommended.

## Appendix

Multimedia Appendix 1 Parameter estimates from linear mixed effects models examining the association between commercial trackers and ActiGraph (steps, moderate-to-vigorous physical activity) and SenseWear (total sleep time).

## Abbreviations

API: application programming interface
eHealth: electronic health
ICC: intraclass correlation coefficient
MAPE: mean absolute percentage error
MVPA: moderate-to-vigorous physical activity
PA: physical activity
SB: sedentary behavior
SES: socioeconomic status
TST: total sleep time
