# Supplementary material for: Low-Cost Consumer-Based Trackers to Measure Physical Activity and Sleep Duration Among Adults in Free-Living Conditions: Validation Study
Source: JMIR Mhealth Uhealth. 2020 May 19;8(5):e16674. doi: 10.2196/16674 (PMC7268004; doi:10.2196/16674)
Supplement: Multimedia Appendix 1 [file mhealth_v8i5e16674_app1.docx]

Multimedia Appendix 1. Parameter estimates from linear mixed effects models examining the association between commercial trackers and ActiGraph (steps, MVPA) and SenseWear (TST).

|  | **Steps** |  | **MVPA^a^** |  | **TST^b^** |  |
| --- | --- | --- | --- | --- | --- | --- |
|  | **B** | **95% CI** | **B** | **95% CI** | **B** | **95% CI** |
| Geonaut | 1.03^c^ | 0.90-1.17 |  |  | 0.05 | -0.26-0.35 |
| iWown | 0.75^c^ | 0.58-0.92 |  |  | 0.57^c^ | 0.28-0.85 |
| MyKronoz | 0.72^c^ | 0.61-0.84 |  |  | 0.55^d^ | 0.12-0.99 |
| Nokia | 0.71^c^ | 0.52-0.90 | -0.26 | -1.20-0.68 | 0.37^d^ | 0.15-0.60 |
| VeryFit | 0.82^c^ | 0.69-0.94 |  |  | 0.64^d^ | 0.23-1.06 |
| Xiaomi | 0.86^c^ | 0.76-0.96 | 0.16 | -0.05-0.37 | 0.34 | -0.13-0.80 |
| Fitbit | 0.83^c^ | 0.79-0.87 | 0.41^b^ | 0.36-0.47 | 0.58 ^c^ | 0.47-0.70 |

^a^ moderate-to-vigorous physical activity

^b^ total sleep time

^c^ P<.001.

^d^ P<.05.
